# The mitochondrial genome of the bone-eating worm *Osedaxrubiplumus*(Annelida, Siboglinidae)

**DOI:** 10.1080/23802359.2020.1772680

**Published:** 2020-06-01

**Authors:** Yadong Zhou, Yuanning Li, Hong Cheng, Kenneth M. Halanych, Chunsheng Wang

**Affiliations:** a Key Laboratory of Marine Ecosystem Dynamics, Second Institute of Oceanography, Ministry of Natural Resources, Hangzhou, China; b Department of Biological Sciences, Vanderbilt University, Nashville, USA; c Department of Biological Sciences, Auburn University, Auburn, AL, USA; d State Key Laboratory of Satellite Ocean Environment Dynamics, Hangzhou, China; e School of Oceanography, Shanghai Jiao Tong University, Shanghai, China

**Keywords:** Osedax, mitogenome, Southwest Indian Ridge, East Pacific

## Abstract

*Osedaxrubiplumus*(Annelida, Siboglinidae)uses heterotrophic bacteria to feed onvertebrate carcasses and is currently found in the Pacific, Antarctic and Indian Ocean.Here, we report its nearly complete mitochondrial genomes assembled for 2 individuals, one from the East Pacific and the other from the Southwest Indian Ocean. Recoveredmitogenomes were 15591 and 15972 bp in length, with both consisting of 37 typical metazoan mitochondrial genes. All genes were transcribed from the same strand, and arranged in the same order as the other siboglinids, revealing conserved gene arrangement withinSiboglinidae. Phylogeneticanalysis of 13 protein coding genes confirms the placement of *Osedax*sister to the Vestimentifera+*Sclerolinum* clade.

The bone-eating worms, *Osedax* spp., are specialists thriving in chemosynthetic ecosystems formed by whale falls and other vertebrate carcassesthrough out the world’soceans (Amon et al. 2014; Rouse et al. 2018; Zhou et al. 2020). They aresiboglinidannelidsand recent analyses (Li et al. 2015, 2017) placed them as sister to the Vestimentiferan + *Sclerolinum* clade.Although the phylogenetic position is well understood, the mitogenome has not been well explored in *Osedax*(Li et al. 2015) and its gene arrangement is still unknown. Interestingly, all sequenced siboglinidmtDNA shared the same gene arrangement (Li et al. 2015). Thus,a wider taxon sampling is needed to better explore the mitogenomein *Osedax*.

Specimens of *O. rubiplumus*were collected fromEast Pacific margin (43°54.52′N; 125°10.29′W, 1560 m) and Southwest Indian Ridge (49°38.685′E, 37°47.013′S, 2908 m), which have been deposited in Auburn University (vouch number: AUMNH 46876) and the Repository of the Second Institute of Oceanography, MNR (vouch number: RSIO49bone_ind) respectively. One individual from each sampling site was used forgenomic DNA extraction, sequencing, assembling and gene annotationfollowing the methods described in Li et al. (2015) and Zhou et al. (2019) respectively. A maximum likelihood (ML) analysis based on concatenated alignments of the amino acid sequences of the 13 PCGs was conductedin IQtree 1.6.10 (Trifinopoulos et al. 2016) with substitution model for each individual gene or partition determined by the program automatically.

Twomitogenomes of *Osedaxrubiplumus* (GenBank accession numbers: MT108936 and MT108937, 15591 and 15972 bp in length respectively) contains 13 PCGs, 2rRNA genes and 22 tRNA genes. Consistent with other siboglinidmitogenomes, the 13 PCGs use ATG as the start codon, and a combination of TAA, TAG and T as stop codon. All genes are transcribed from the same strand, and arranged in the sameorder asother siboglinids, suggesting conserved gene arrangement in the family (Li et al. 2015).

Previous study revealed four major lineages in Siboglinidae: Vestimentifera, Frenulata, *Sclerolinum*, and *Osedax* (Rouse et al., 2004; Halanych 2005). Using the 13 PCGs of *Osedaxmucofloris* mitochondrion, Li et al. (2015) Li et al. (2017) suggested that *Osedax* is genetically closer to the Vestimentifera + *Sclerolinum* claderather than to Frenulata. The maximum likelihoodanalysis in the present study robustly supports the sister relationship between the *Osedax* and Vestimentifera + *Sclerolinum* clade (Figure 1).

**Figure 1.**
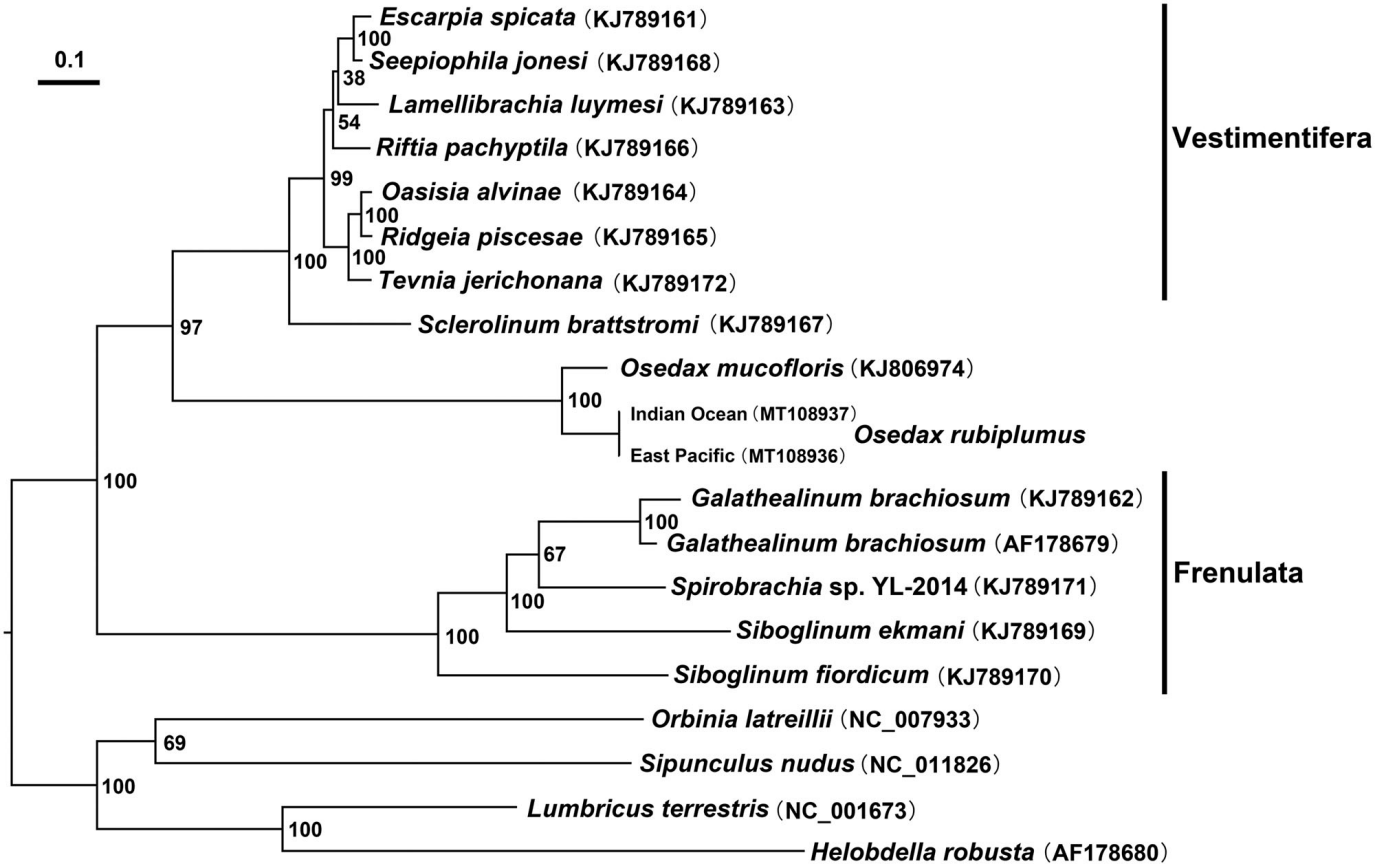
Maximum likelihood (ML) analysis based on the concatenated amino acid (AA) sequences of 13 PCGs. ML bootstrap values are indicated at each node. *Helobdellarobusta*, *Lumbricusterrestris*, *Orbinialatreillii* and *Sipunculusnudus* serve as the outgroup.

## Data Availability

All sequencesgenerated or used in the present study are deposited in NCBI GenBank (https://www.ncbi.nlm.nih.gov) and the accession numbers are detailed in Figure 1.
